# Twists and turns

**DOI:** 10.7554/eLife.32709

**Published:** 2017-11-28

**Authors:** Emily S Noël, Jeroen Bakkers

**Affiliations:** 1 Department of Biomedical ScienceUniversity of SheffieldSheffieldUnited Kingdom; 2 Hubrecht Institute - Royal Netherlands Academy of Arts and Sciences (KNAW), University Medical Centre UtrechtUtrechtNetherlands

**Keywords:** left-right patterning, heart morphogenesis, computer modelling, 3D shape, None

## Abstract

Computational modelling of the heart tube during development reveals the interplay between tissue asymmetry and growth that helps our hearts take shape.

**Related research article** Le Garrec JF, Domínguez JN, Desgrange A, Ivanovitch KD, Raphaël E, Bangham JA, Torres M, Coen E, Mohun TJ, Meilhac SM. 2017. A predictive model of asymmetric morphogenesis from 3D reconstructions of mouse heart looping dynamics. *eLife* **6**:e28951. doi: 10.7554/eLife.28951

While humans appear to display left-right symmetry when viewed from the outside, our internal organs are not positioned symmetrically within our body and some of them also exhibit intrinsic left-right asymmetries. These asymmetries develop very early during embryonic development, and understanding how they arise and change over time is a central challenge in developmental biology.

The heart is the first organ to display left-right asymmetries, and is a key example of an organ for which asymmetric development is critical for its function. During the early stages of cardiac development, the heart tube – the structure that develops into the heart – changes shape from a simple linear tube to a more complex structure with a rightward helical loop (Patten, 1922). This process, which is known as heart looping, is crucial for ensuring that the future chambers of the heart are aligned properly, and defects during the looping process can result in congenital heart disease and other serious heart conditions (Männer, 2009).

Although the transition from a linear to a looped heart tube appears to be relatively simple, the various mechanisms that contribute to this transition, and the interactions between them, are highly complex. Two of the key factors driving the morphogenesis of the heart are the growth of the heart tube and laterality (that is, differences between cells that originate on the left and right side of the body, including heart cells). Many studies have shown that the formation of the rightward helical loop in the higher vertebrates is partly under the control of signalling pathways that are more active on one side of the body than the other side (Campione and Franco, 2016). At the same time, the heart tube also undergoes a period of growth, driven by the ingression of cells from the surrounding tissue into both ends of the tube (Stalsberg, 1969). This ingression of cells helps the heart tube to increase in length by a factor of four in less than a day. Now, in eLife, Sigolène Meilhac of the Pasteur Institute and *Imagine* Institute in Paris and co-workers – including Jean François Le Garrec as first author – report how they have used a combination of computational models and in vivo observations to explore the interactions between laterality and growth during heart looping in mice (Le Garrec et al., 2017).

Le Garrec et al. used high-resolution microscopy to show that the early heart tube exhibits rotational asymmetry at the arterial pole (the pole through which blood leaves the heart tube; Figure 1), and that it is positioned asymmetrically across the embryonic midline. The identification of this rotational asymmetry in the mouse heart tube prior to looping reveals new similarities with heart development in other model organisms such as chick (rotational asymmetry at the arterial pole; de la Cruz and Markwald, 1998) and zebrafish (asymmetrical rotation and positioning of the heart tube; Chen et al., 1997; Smith et al., 2008). The researchers also used lineage tracing to demonstrate that ingression and proliferation of cardiac progenitor cells at the venous pole (the pole through which blood enters the heart tube) is asymmetric: this suggests that the growth of the heart tube and the asymmetries at both poles act together to drive both the leftward displacement and the rightward looping of the heart.

**Figure 1.**
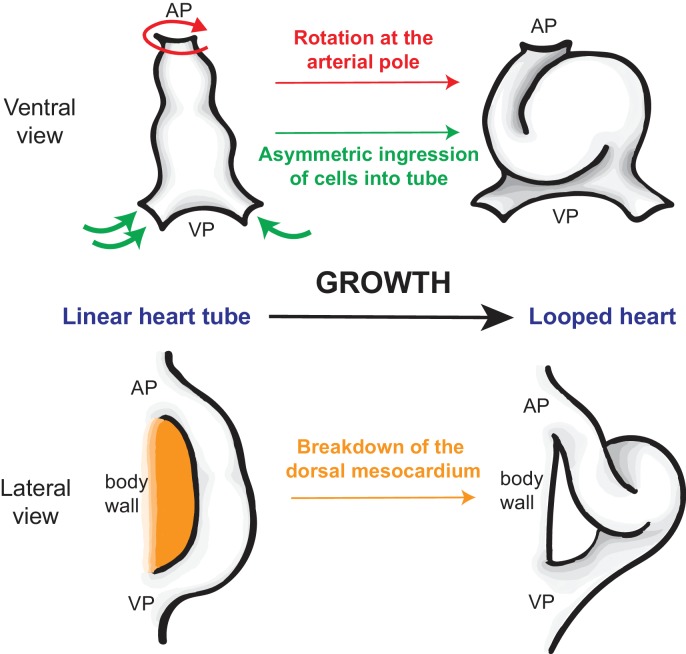
How the heart tube forms a helical loop. The heart develops from a primitive structure called the heart tube (top left). As the heart tube increases in length, the top of the tube (known as the arterial pole; AP) and the bottom of the tube (venous pole; VP) remain in position, so the tube buckles and twists to form a helical loop (top right), which undergoes further development to form a mature heart (not shown). The loop is always in a rightward direction (which is on the left in this ventral view). Le Garrec et al. show that opposing rotations at the two poles, together with the asymmetric ingression of cells into the heart, ensures that the loop is to the right. Simultaneously, the dorsal mesocardium, which attaches the heart tube to the body wall of the embryo (orange; bottom left), breaks down as the heart grows. This releases the heart tube so that it is only attached at the arterial and venous poles, allowing the helical loop to form (bottom right).

To better understand these processes and interactions, Le Garrec et al. used a sophisticated computational model that had previously been employed to model the shapes of flowers (Green et al., 2010). Combining morphological observations with the computational model allowed the researchers to see how varying the input parameters (such as the physical distance between the two poles of the heart, the asymmetric growth at the poles, and how the heart tube is connected to the surrounding tissue) led to subtle changes in morphology of the looped heart tube. The model developed by Le Garrec et al. found that the normal breakdown of the tissue between the heart tube and the body wall of the embryos (the dorsal mesocardium) had an important role in helical looping. The researchers then confirmed this prediction in experiments with mouse mutants in which the dorsal mesocardium does not break down as it should.

The detailed modelling and analyses undertaken by Le Garrec, Meilhac and colleagues – who are based in France, Spain and the United Kingdom – provides a new benchmark for how we assess and define looping morphogenesis defects in genetic mutants. The drive towards a better understanding of complex morphogenetic behaviours will promote more comprehensive analyses of heart defects in genetic mutants, and will help us to refine our ideas of how this elegant process is controlled.

Recent studies by Robert Kelly’s group at Aix-Marseille University have shown that the cells surrounding the heart which ingress to allow the heart to grow, exhibit different regionalised tension which is linked to heart growth and looping (Francou et al., 2017). Together with the studies from Le Garrec et al., this suggests that the coordination of specific biomechanical forces and cell behaviours in different parts of the heart all work together to help the heart loop.

The results presented by Le Garrec et al. mean that we now better understand the biomechanical forces that can drive the complex process of cardiac looping. However, the the question of how these cell and tissue asymmetries are achieved in the developing embryo remain unanswered. Is a recently identified cell intrinsic asymmetry in the cytoskeleton (Tee et al., 2015) responsible for the asymmetric biomechanical forces, or is the laterality pathway providing asymmetric cues to the cells, or is it a combination of both? Answering this question will ultimately help us to better define all the processes, both within the early embryo and within the heart itself, that contribute to shaping our hearts.
